# Survey on the Presence of *Malassezia* spp. in Healthy Rabbit Ear Canals

**DOI:** 10.3390/pathogens9090696

**Published:** 2020-08-25

**Authors:** Roberta Galuppi, Benedetto Morandi, Silvia Agostini, Sara Dalla Torre, Monica Caffara

**Affiliations:** Department of Veterinary Medical Sciences, Alma Mater Studiorum, University of Bologna, Tolara di Sopra 50, 40064 Ozzano Emilia, Bologna, Italy; benedetto.morandi2@unibo.it (B.M.); silvia.agostini4@studio.unibo.it (S.A.); sara.dallatorre@studio.unibo.it (S.D.T.); monica.caffara@unibo.it (M.C.)

**Keywords:** *Malassezia*, phylotype 131 yeast, lagomorph, rabbit, ear canal skin

## Abstract

*Malassezia* spp. have rarely been reported in rodents and lagomorphs. In 2011, *Malassezia cuniculi* was described in two rabbits. Further microscopic studies showed *M. cuniculi*-like yeasts in more than 50% of samples from rabbits’ ear canals, but no isolation was made. The present study details the presence of *Malassezia* spp. and tries to typify it from ear canals of healthy rabbits. Seventy-eight half-breed rabbits from rural farms and 98 companion dwarf rabbits from northern Italy were considered. A first attempt to screen ear swabs was performed by microscopic and cultural examination on Sabouraud Glucose Agar (SGA), modified Dixon Agar (mDA) and Leeming and Notman Agar (LNA). Additionally, ear swabs from eight further microscopically positive rabbits for *M. cuniculi*-like cells, were used for both isolation on LNA medium and nine of its variants and for DNA extraction, PCR and sequencing. The microscopic observation of the swabs of the screened 168 rabbits highlighted the presence of yeasts in one or both of the external ear canals of 98 rabbits (58.3%). Rabbits used for meat production were more frequently diagnosed positive than pet rabbits (P = 0.001), and young ones were more often positive compared to rabbits older than 3 months (P = 0.027). No yeast growth was observed in culture. From the eight selected rabbits, *Malassezia* isolation failed both on LNA and on the modified mediums. Sequences of ~300 bp fragments of 18s rDNA, obtained by PCR from swabs, showed 99.9% identity with *Malassezia* phylotype 131 described from human ear canals. As *Malassezia*-like yeasts have been observed in more than half of the examined population, its colonization of ear meatus can be considered as physiological in rabbits. The results outline how much remains to be discovered on *Malassezia* as a component of the skin mycobiota of rabbits and that the use of the culture examination alone is not the best choice to detect *Malassezia*-like yeasts in rabbits.

## 1. Introduction

The genus *Malassezia*, belonging to the class Malasseziomycetes of Ustilaginomycotina (Basidiomycota, Fungi) [1], includes lipophilic yeasts being both commensal microorganisms and pathogens on the skin of euthermic animals. The presence of *Malassezia* spp. is rarely reported in rabbits. In 1985, Dufait [2] isolated *Malassezia pachydermatis* from the hair of several rabbits. Later, in 1994, Guillot et al. [3] did not detect this yeast in lagomorphs, rodents and insectivores neither by microscopic examination nor by culture; they suggested that the absence of *Malassezia* in the auditory conduit of these animals could be related to the small amount of cerumen, an essential source of lipids for these yeasts. After that, *Malassezia* spp. has been considered to not affect lagomorphs. Only one case of dermatitis is associated with *Malassezia* spp. growing on Sabouraud dextrose agar, therefore, most likely *M. pachydermatis*, was recently described in Peru [4]. In 2004, Radi [5] described the presence of budding yeast referable to *Malassezia* spp. morphologically different from *M. pachydermatis* in histological sections of rabbits’ skin with sarcoptic mange. Cabañes et al. [6], in 2011, carried out a research on 11 healthy New Zealand rabbits. The authors isolated a *Malassezia*-like yeast with round budding cells from the external ear canal and inguinal skin of two subjects, growing only on Leeming and Notman Agar (LNA) but neither on modified Dixon Agar (mDA) nor on Sabouraud Glucose Agar (SGA). After morphological, physiological and molecular characterization, the authors described this isolate as a new species named *Malassezia cuniculi*. Subsequently, Quinton et al. [7], in a cytological survey on external auditory meatus of 146 healthy pet rabbits, reported the presence of unipolar budding cells morphologically referable to *Malassezia* yeast in 54% of the samples, speculating that it was probably *M. cuniculi* without performing cultural or molecular tests.

This study aims to survey the presence of *Malassezia* spp. in ear canals of healthy rabbits through cytological and cultural examination, assessing correlation with gender, age, breeding, housing and feeding systems. Additionally, further cultural and molecular assays were performed to typify the involved yeasts.

## 2. Materials and Methods

The study was performed on 176 rabbits, either for meat production or for companionship, from northern Italy. No invasive procedures were used—only ear swabs in agreement with the owners— and no personal data were collected. Included animals were in a good state of nutrition, showed neither cutaneous alterations nor signs of otitis.

A first screening was performed on 168 rabbits, 70 mixed breed from rural farms and 98 companion dwarf rabbits. Samples were collected from both the external ear canals of each animal using swabs soaked in phosphate buffered saline (PBS) containing 0.1% Tween 80 [6] (Sigma-Aldrich, Milano, Italy). All samples were inoculated onto SDA (BBL-BD), mDA and Leeming and LNA, all included 0.05% chloramphenicol [6]. The plates were incubated at 32 °C and examined daily for 20 days. All swabs were also rolled over the surface of microscopic slides and the smears were stained with May Grunwald-Giemsa.

Subsequently, a further eight mixed breed rabbits cytologically positive for *Malassezia cuniculi*-like yeasts in both external ear canals were selected from a rural farm. From each animal, a further five swabs were used to collect yeasts: One was soaked in Tris-EDTA (TE) buffer and used for molecular analysis, four were soaked in PBS containing 0.1% Tween 80, and used for cultures. The culture medias used were LNA and nine of its variants, empirically created modifying LNA mainly by adding various lipid sources (Table 1). The plates were inoculated by streaking the swabs, then incubated at 32 °C and examined daily for 20 days.

DNA extraction from swabs was performed using PureLink^®^ Genomic DNA Mini Kit (Life Technologies, Carlsbad, CA, USA) according to manufacturer’s instruction for yeast cells, slightly modified as follows: Each swab was put in 500 µl Zymolase Buffer with 15 U Lyticase (Sigma-Aldrich, Milano, Italy) and incubated at 37 °C for one hour, shaking periodically, then centrifuged at 3000 × *g* for 10 min at room temperature to pellet the spheroplast. As a positive control, one ear swab from a dog positive to *M. pachydermatis* was used, whereas a sterile swab without any skin contact submitted to the same procedures was used as negative one.

The primers Mal-F 5′ GTC CGC GTT GTA ATC TCG AGA CGT and Mal-R 5′ TGG CTG ACT TCA AGC GCT TCC CTT, highly specific for *Malassezia* [8], were used to amplify a fragment of ~300 bp of 26S rRNA of *Malassezia* spp., included into the D1/D2 region.

For sequencing, bands were excised, purified by NucleoSpin Gel and PCR clean-up (Mackerey-Nagel, Düren, Germany), sequenced with an ABI 3730 DNA analyzer at StarSEQ GmbH (Mainz, Germany) and assembled with Vector NTI Advance 11 software (Life Technologies, Carlsbad, CA, USA). Blast analysis was performed and multiple sequence alignments with *Malassezia* spp. sequences, retrieved from GenBank, were constructed using BioEdit 7.2.5 [9]. The sequences obtained were deposited in GenBank under accession numbers MF983546-53.

Pearson’s chi-square test was used for the comparison of gender, age, breeding, housing and feeding systems as potentially affecting the presence of *Malassezia* spp. Results were considered significant when P ≤ 0.05. Stata Statistical Software (Release 15, College Station, TX: StataCorp LLC) was utilized for the descriptive statistical analysis.

## 3. Results

Table 2 shows the distribution of different housing conditions and rabbits’ breeding by sex, age and feeding methods. Overall, the screened population accounted for 168 rabbits, of which 70 (41.7%) were used for meat production and the remaining 98 (58.3%) were companion animals. As for gender, 83 (49.4%) rabbits were males and 85 (50.6%) females. Furthermore, 72 (42.9%) were up to 3-months-old and 96 (57.1%) older than 3-months-old. Eighty-six (51.2%) were hosted in-home being free to roam, 69 (41.1%) were caged and 13 (7.7%) hosted in cages in a rural environment. As for the different feeding practices, 131 (78%) had mixed feeding (hay, pellet, bread and vegetables) and the remaining 37 (22%) were exclusively fed pellet. Sex distribution was not affected by different housing methods (*P* = 0.967) and by involved category of rabbits (*P* = 0.419), on the contrary age was dependent on such categories (*P* < 0.001). 

**Figure 1:**
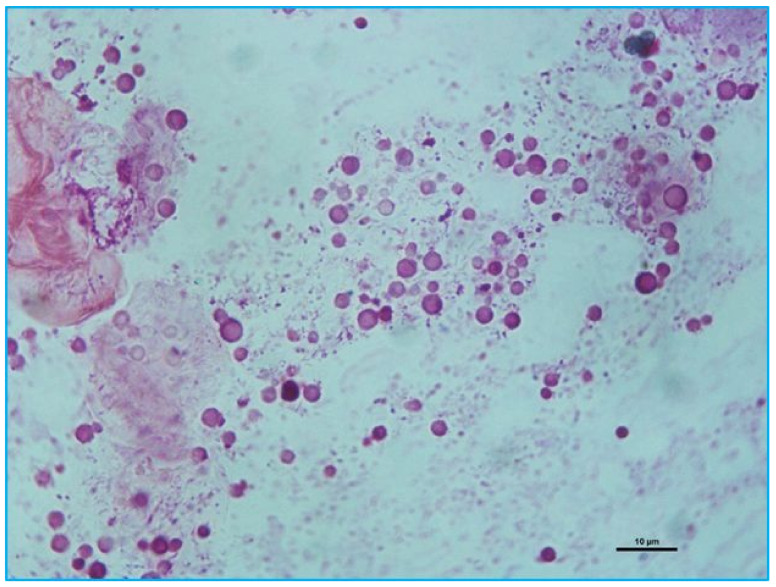
May Grunwald-Giemsa stain of smear from an ear swab showing the presence of *Malassezia cunicoli*-like cells.

*Malassezia*-like yeasts were cytologically observed from at least one ear conduct of 98 (58.3%) rabbits (65 bilateral, 33 unilateral). The yeasts, usually observed in cluster not uniformly distributed, were rounded, 2–4 µm in diameter, with buds formed in a monopolar pattern on narrow bases (Figure 1), consistent with the description of *M. cuniculi* [6]. No yeast growth in the three cultures medias employed as screening was observed.

Considering the two categories, meat production and companion intent, *Malassezia*-like yeasts were cytologically observed with significantly higher frequency in meat producing rabbits (72.9%) compared to pet rabbits (48%) (χ2 = 10.41; *P* = 0.001). Furthermore, *Malassezia* was more often bilaterally detected in meat production systems compared to companion animals, 76.5% and 56.5%, respectively (χ2 = 4.35; *P* = 0.037). As for sex, 67.1% of the females and 49.4% of the males harbored yeasts (χ2 = 5.39; *P* = 0.02). Determination of the Pearson χ2-test showed age patterns, where rabbits 3-months-old or younger were more often cytologically diagnosed positive for *Malassezia*-like yeasts compared to rabbits older than 3 months, 68.1% and 51%, respectively (χ2 = 4.9; *P* = 0.027). Additionally, differences among housing methods were found (χ2 = 6.747; P = 0.034), since *Malassezia*-like yeasts were diagnosed in 12 (92.3%) rabbits from cages in a rural environment, in 39 (56%) rabbits permanently fenced in and finally in 47 (54.6%) out of the rabbits free to roam in-home. Considering feeding methods, rabbits fed only with pellet were more frequently diagnosed positive for yeasts (83.8%) compared to mixed feeding rabbits (51.1%), this difference showed a statistical significance to the Pearson χ2-test (χ2 = 12.65; P < 0.001). Age class and feeding methods were strictly dependent on housing and breeding methods (P < 0.001). Results are summarized in Table 3. 

**Figure 2:**
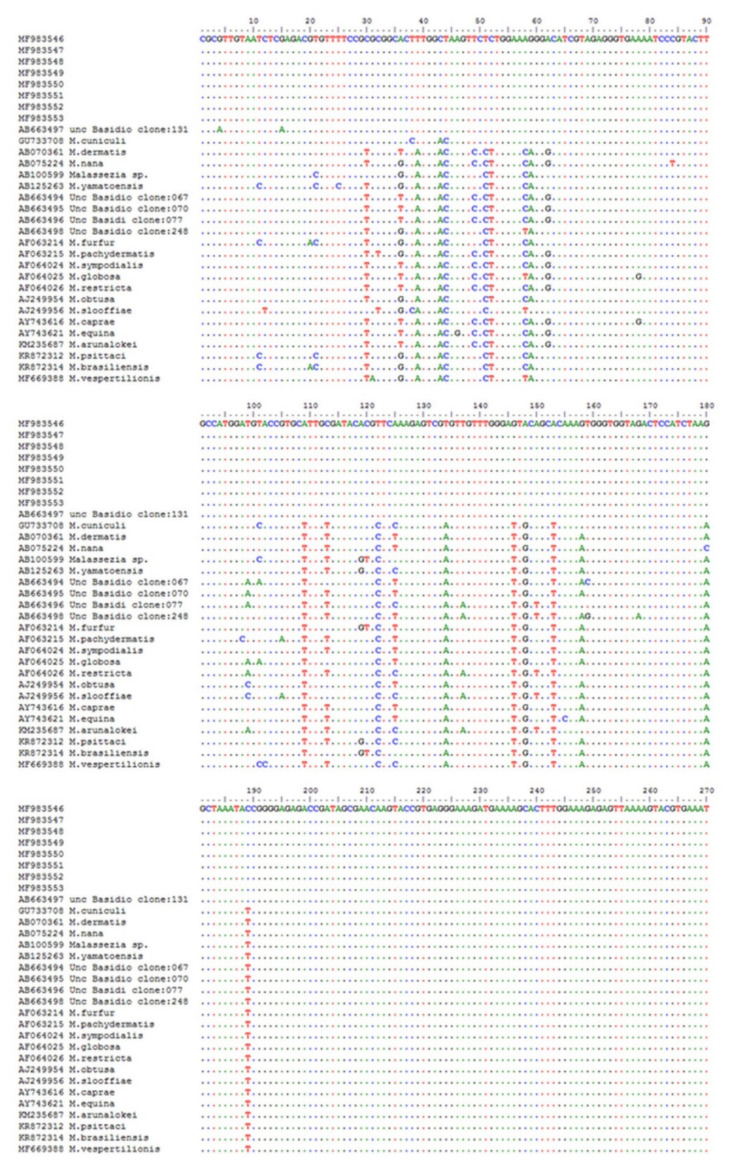
Alignment of 26S rDNA including variable region D1-D2. GenBank acc. n. MF983546-53= *Malassezia* sp. sequences generated in the present study.

**Figure 3:**
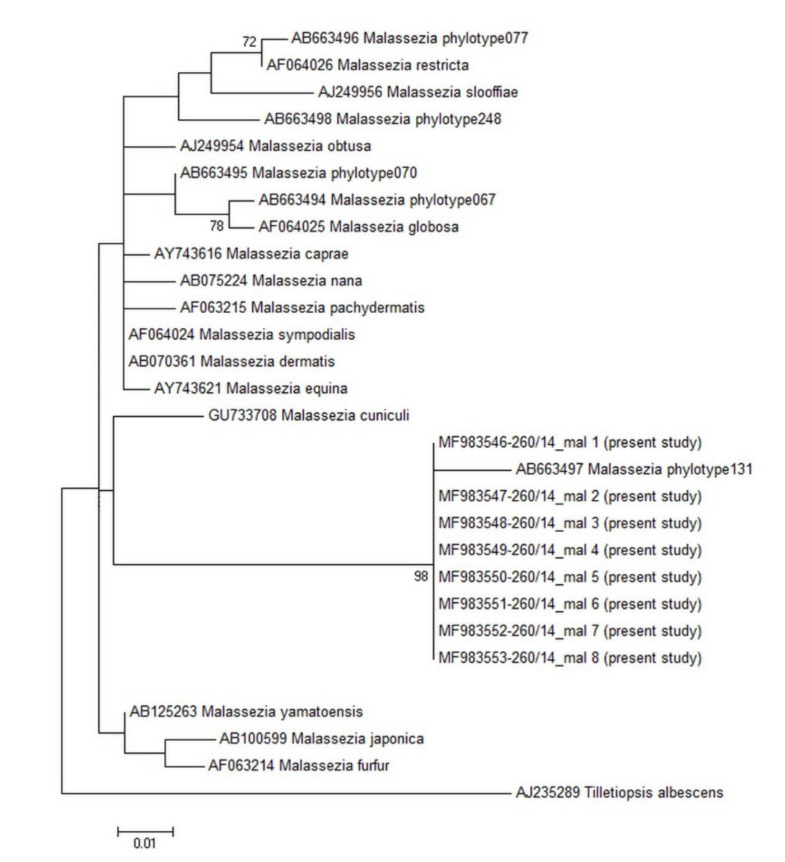
Evolutionary history inferred by maximum likelihood method based on the Kimura 2-parameter model. The analysis involved 29 nucleotide sequences for a total of 234 positions in the final dataset. Bootstrap values (>70%, 1000 replicates) are shown next to the branches.

Concerning the culture test performed on the eight selected rabbits cytologically positive, no ear swab yielded colonies on LNA, and none of the modified medium used in the present study allowed the yeast growth. All the eight swabs from these rabbits and the one from dog, used as a positive control, tested positive with PCR, giving a band at around 300 bp. The sequences obtained from all rabbits were identical to each other and showed a BLAST identity of 99.9% with uncultured Basidiomycota clone 131 (AB663497), later on named *Malassezia* phylotype 131, isolated from human external auditory canal, not growing on LNA medium [10]. Our specimens differed from the phylotype 131 by 2 transition G/A (Figure 2). The phylogenetic tree (Figure 3) showed our strains forming a well-supported (98%) cluster together with the phylotype 131, which was clearly separated from the rest of the *Malassezia* species, as already reported [10].

## 4. Discussion

With the exception of *M. pachydermatis*, considered a single non-lipid dependent species, new lipid-dependent species have been described over time, not only in humans but also in animals. The most recently described *M. brasiliensis*, *M. psittaci* [11], *M. arunalokei* [12] and *M. vespertilionis* [13], led to 18 being the number of species belonging to this genus, many of which are considered host specific.

Unlike other mammals, in the past *Malassezia* spp. was rarely described in rabbits [2,3] and, to our knowledge, only one case of dermatitis has been associated to *Malassezia* spp. growing on SDA, therefore most likely *M. pachydermatis*, was described in the literature [4]. *M. cuniculi*, characterized by growing only on LNA medium, was isolated and described in Spain from only two rabbits by Cabañes et al. [6], who were the first to report a species-specific *Malassezia* in rabbits. In the present study, we have shown that *M. cuniculi*-like yeasts are frequently detected in healthy rabbit ear swabs (58.3%) in agreement with Quinton et al. [7], who reported, by performing cytological examination, a frequency around 53%. These authors stressed that the discrepancies with previous studies [3] that did not find Malassezia in rabbits, could be due to the nature of population studies (pet vs breeding/meat production). Conversely, in the present study rabbits used for meat production were more frequently diagnosed positive than pet rabbits, and more often bilaterally, suggesting a higher colonization of this category. Young animals were more often positive compared to ones older than 3 months; similarly, rabbits fed only with pellet had higher frequency of positive smears. Quinton et al. [7] observed no differences related to the age classes, nevertheless, our results showed that age class and feeding are strictly dependent on housing and breeding methods (Table 2), possibly leading to a confounding effect of age on *Malassezia*-like yeast detection. 

The frequent finding of *Malassezia cuniculi*-like yeasts in cytological examination on healthy rabbits, suggests that these yeasts are common commensal in these animals. However, there are few studies describing the presence of *Malassezia* spp. and its role in rabbits. *Malassezia* is not usually considered among the causes of dermatitis in rabbits [14]. Only one case of dermatitis, probably due to *M. pachydermatis* due to its ability to grow on SDA, has been described [4]. A retrospective study about skin diseases in pet rabbits carried out at the University of California at Davis, USA [15] highlights alopecia and otitis as common skin lesions, despite an etiological diagnosis is not always defined and the diagnostic methods used are also not described (apart from bacterial culture). However, these studies are previous to the description of the fastidious *M.cuniculi*. 

These rare diagnoses of *Malassezia*-like yeasts in rabbits may be explained by several reasons: i) *Malassezia* had not been considered in rabbits, ii) low number of animals checked, iii) the diagnostic methodologies applied. Although, in mycology, culture methods are usually more sensitive than microscopic examination, the use of fungal cultures may only have produced an underestimation of *Malassezia* presence in rabbits, associated or not to dermatitis. 

Although the LNA has been described as the suitable medium for the growth *M. cuniculi*, we have not observed any growth using this medium, as well as using mDA or SDA. This supports the hypothesis that some variants could be present, likely related with geographical origin [16], having different metabolic requirements. This was demonstrated in *M. pachydermatis*, which shows many variants with different morphological, biochemical and molecular characteristics [17,18,19,20]. 

For this reason, in this study, in order to obtain isolates from the ear of cytologically positive rabbits, nine LNA variants, empirically created modifying LNA mainly by adding various lipid sources (Table 1), were used. None of the modified medium used was able to allow the yeast growth. As pointed out by Cabañes et al. [21], the exact nutritional requirements of *Malassezia* species in culture are yet to be fully determined and this hinders the study of these yeasts. Our findings reveal that the sensitivity of the culture system for the detection of *Malassezia*-like yeasts can be equal to zero, even though several mediums are used.

In order to overcome this problem, DNA extraction directly from the swab could be a useful tool. To our knowledge, molecular detection of *Malassezia* directly from biological samples has been described in the literature, basically in the human field [10,22,23,24], using fungal universal primers. In particular, Jung et al. [24], evaluating the nasal vestibule microbiota in patients with allergic rhinitis, obtained sequences referable to *M. cuniculi* in a patient, in addition to other more frequent species such as *M. restricta* and *M. globosa* (predominant), *M. slooffiae*, *M. sympodialis*, *M. dermatis* and *M. pachydermatis*.

In the animal field, the abundant fungal flora contaminant or resident over the skin, can complicate the direct amplification of *Malassezia* using universal primers. Recently, Puig et al. [25] developed a fast qPCR amplifying the ß-tubilin gene, directly from dog-ear swabs, using primers specific for *M. pachydermatis*. 

The primers used in our study [8] are highly specific for the genus and facilitate the amplification of *Malassezia* spp. directly from the ear swabs. The amplified fragment, even though consisting of only ~300 bp, is included into the D1/D2 domain of the 26S rRNA. This domain could be used for the identification of most basidiomycetous yeast species, although the internal transcribed spacer region is required to distinguish closely related species [26]. The sequences obtained from all the eight rabbits examined were identical to each other; they differed from *M. cuniculi* ones and showed 99.9% identity with *Malassezia* phylotype 131, a strain not growing on LNA and detected only by PCR by Zhang et al. [10] from the human external auditory canal. 

The short sequences obtained are not suitable for inferring accurate phylogenetic relationships if used as the only marker, therefore the tree obtained herein was only aimed at showing the relationship with the other *Malassezia* yeasts. Nevertheless, the cluster in which our sequences are included is well supported (98%). Differences with other *Malassezia* spp. can support the hypothesis of distinct variants having different metabolic requirements and would explain troubles in obtaining isolates by culture media. 

In conclusion, since *Malassezia*-like yeasts have been observed in more than half of the healthy examined population, its colonization of ear meatus can be considered as physiological in rabbits, being part of the normal skin microbiota. In the future, the control for *Malassezia* based on the rabbits’ physiological status, and the presence of ear or skin disease, could be useful to clarify if *Malassezia* is able to play a pathogenic role in this species. Additionally, it appears clear that the use of culture examination alone is not the best choice to detect *Malassezia*-like yeasts in rabbits. 

The strain here reported certainly cannot be considered a new species, due to the lack of isolation and further genetic information, however, the results obtained outline how much remains to be discovered on *Malassezia* as a component of the rabbits mycobiota. Thus, we strongly believe that our results might be useful for further future studies. 

## Figures and Tables

**Table 1:** Composition per L of deionized water of Leeming and Notman Agar (LNA) and the other media used. The media were empirically created modifying the LNA mainly by adding various lipid sources. All the media contained 0.05% of chloramphenicol ^2^.

Component	LNA	Medium A	Medium B	Medium C	Medium D	Medium E	Medium F	Medium G	Medium H	Medium I
Peptone^1^	10 g	10 g	**6 g**	10	10 g	10 g	10 g	10 g	10 g	10 g
Glucose^2^	5 g	5 g	5 g	5 g	5 g	5 g	5 g	5 g	5 g	5 g
Yeast extract^1^	0.1 g	0.1 g	**0.5 g**	0.1	0.1	0.1 g	0.1 g	0.1 g	0.1 g	0.1 g
Bile bovine^2^	4 g	4 g	4 g	4 g	4 g	**20 g**	4 g	4 g	4 g	**20 g**
Glycerol^2^	1 mL	-	1 mL	1 mL	1 mL	1 mL	1 mL	1 mL	1 mL	1 mL
Glycerol monostearate^3^	0.5 g	0,5 g	0.5g	0.5	0.5 g	0.5 g	0.5 g	0.5 g	0.5 g	0.5 g
Tween 60^2^	0.5 mL	0.5 mL	0.5 mL	0.5	0.5 mL	0.5	-	-	-	-
Tween 40^2^	-	**0.5 mL**	-	**0.5**	**0.5 mL**	-	-	-	-	-
Tween 80^2^	-	-	-	-	-	-	-	**0.5 mL**	-	-
Tween 20^2^	-	-	-	-	-	-	-	-	**0.5 mL**	-
Cholesterol^2^	-	-	-	**10 mg**	-	-	-	-	-	-
Boiling extract of ear rabbit^6^	-	-	-	-	**10 mL**	-	-	-	-	-
Oleic acid^2^	-	-	-	-	-	-	**0.5 mL**	-	-	-
Olive oil^4^	-	-	-	-	-	-	-	-	-	**10 mL**
Malt extract^1^	-	-	**3 g**	-	-	-	-	-	-	-
Whole-fat cows milk^5^	10 mL	10 mL	10 mL	10 mL	10 mL	10 mL	10 mL	10 mL	10 mL	10 mL
Agar^1^	12 g	12 g	12g	12 g	12 g	12 g	12 g	12 g	12 g	12 g

Source of Material: ^1^BD, Le Pont of Claix, France; ^2^Sigma-Aldrich, Milano, Italy; ^3^Fagron, Bologna, Italy; ^4^Monini, Spoleto, Italy; ^5^Granarolo, Bologna, Italy. ^6^It was obtained boiling 2 rabbit ears in deionized water for 1 h, allowing to cool and taking 10 mL of the most superficial layer of the cooking liquid.

**Table 2:** Distribution of different housing methods and rabbits’ categories by sex, age and feeding.

		Housing Methods			Breeding	
		Home (%)	Cage (%)	Rural (%)	Meat (%)	Companion (%)
Sex*	**Males**	43 (50)	34 (49.3)	6 (46.1)	32 (45.7)	51 (52)
	**Females**	43 (50)	35 (50.7)	7 (53.9)	38 (54.3)	47 (48)
**Age**	**≤ 3months**	20 (23.3)	41 (59.4)	11 (84.6)	52 (74.3)	20 (20.4)
	**> 3 months**	66 (76.7)	28 (40.6)	2 (15.4)	18 (25.7)	78 (79.6)
**Feeding**	**Mixed^a^**	86 (100)	32 (46.4)	13 (100)	33 (47.1)	98 (100)
	**Pellet**	0	37 (53.6)	0	37 (52.9)	0
	**Total**	86	69	13	70	98

* Not dependent on housing methods and breeding systems. ^a^ Mixed: Hay, pellet, bread, vegetables.

**Table 3:** Comparison among predictors and cytologically diagnosed positive rabbits for *Malassezia*-like yeasts.

Predictors	Categories	Percentages (%)	χ^2^ coeff.	P-Value
Production systems	Meat	72.9	10.41	0.001
	Companion	48		
Sex	Female	67.1	5.39	0.02
	Male	49.4		
Age	≤3 months	68.1	4.9	0.027
	>3 months	51		
Housing methods	Home	54.6	6.747	0.034
	Cage	56.5		
	Rural	92.3		
Feeding	Pellet	83.8	12.65	<0.001
	Mixed	51.1		
